# Supplementary material for: Assessing the effectiveness and implementation of a universal classroom-based set of educator practices to improve preschool children’s social-emotional outcomes: Protocol for a cluster randomized controlled type 2 hybrid trial in Singapore
Source: PLoS One. 2023 Sep 20;18(9):e0291723. doi: 10.1371/journal.pone.0291723 (PMC10511079; doi:10.1371/journal.pone.0291723)
Supplement: S1 Fig — (DOC) [file pone.0291723.s002.doc]

Figure. SPIRIT schedule of enrolment, interventions, and assessments

|  | **STUDY PERIOD** | | | | | |  |
| --- | --- | --- | --- | --- | --- | --- | --- |
|  | **Enrolment** | **Allocation** | **Post-allocation** | | | | **Close-out** |
| **TIMEPOINT** | ***Recruitment*** | | ***T1: Baseline Assessment*** | ***T2: Post-training workshop*** | ***T3: Mid-intervention*** | ***T4: Post-intervention*** | ***Analysis & writeup*** |
| **ENROLMENT:** |  |  |  |  |  |  |  |
| **Eligibility screen** | X |  |  |  |  |  |  |
| **Informed consent** | X |  |  |  |  |  |  |
| **Allocation** |  | X |  |  |  |  |  |
| **INTERVENTIONS:** |  |  |  |  |  |  |  |
| ***Intervention Group  (EASEL Approach)*** |  |  | X | X | X | X |  |
| ***Control Group*** |  |  | X |  |  | X |  |
| **ASSESSMENTS:** |  |  |  |  |  |  |  |
| ***Caregiver/child/educator demographic variables*** |  |  | X |  |  |  |  |
| ***Child Self-Regulation & Behaviour Questionnaire*** |  |  | X |  |  | X |  |
| ***Behavior Rating Inventory of Executive Functioning – Preschool*** |  |  | X |  |  | X |  |
| ***Whole Child Panel 2.2*** |  |  | X |  |  |  |  |
| ***Early Childhood Classroom Observation Measure*** |  |  | X |  |  | X |  |
| ***Post-training workshop questionnaire*** |  |  |  | X |  |  |  |
| ***Educator self-assessment*** |  |  |  | X | X | X |  |
| **ANALYSIS & WRITEUP:** |  |  |  |  |  |  | X |
