# Supplementary material for: Assessing the effectiveness and implementation of a universal classroom-based set of educator practices to improve preschool children’s social-emotional outcomes: Protocol for a cluster randomized controlled type 2 hybrid trial in Singapore
Source: PLoS One. 2023 Sep 20;18(9):e0291723. doi: 10.1371/journal.pone.0291723 (PMC10511079; doi:10.1371/journal.pone.0291723)
Supplement: Attachment — Submitted filename: PONE-D-23-00745 Response to Reviewers Jul2023.docx [file pone.0291723.s003.docx]

PLOS ONE Manuscript Response to Reviewers

ID: PONE-D-23-00745

Title: Assessing the effectiveness and implementation of a universal classroom-based set of educator practices to improve preschool children's social-emotional outcomes: Protocol for a cluster randomized controlled type 2 hybrid trial in Singapore

**We thank the reviewers for offering constructive and helpful comments to help improve our manuscript.**

**Please note that the page numbers referred to in the authors’ responses are in reference to pages in the manuscript with tracked changes.**

| **#** | **Reviewers’ Comments** | **Authors’ Response** |
| --- | --- | --- |
| **REVIEWER 1** | | |
| 1 | This protocol has no clearly defined primary outcomes. I understand that the authors have mentioned in the manuscript that “Primary outcomes include educators' teaching practices and their adoption of the EASEL Approach in everyday practice”. Under the “Assessing effectiveness” section, there are several outcomes, which are potentially the outcomes to assess the EASEL approach in everyday practice. Are them all co-primary outcomes? | For clarity, we have amended this statement in the Abstract (p.3) to specify that our primary outcome is educators’ teaching practices, and that their adoption of the EASEL Approach in everyday practice is a secondary outcome, along with acceptability and feasibility of the EASEL Approach and children’s SEB+EF outcomes.  We use a type 2 hybrid study for this trial (Curran et al., 2012). This relatively new classification of design acknowledges that the causal chain to impact includes the service itself and how it is delivered. Type II hybrid studies are used when the effectiveness of the intervention and the effectiveness of implementation strategies/approaches are unknown and are of equal interest.  Our outcomes related to effectiveness are primarily educators’ classroom management, the climate of the classroom, and educators’ instruction (as assessed on the ECCOM), and secondary outcomes are child social, behavioral, emotional regulation, and executive functioning outcomes (as assessed on the BRIEF-P and CSBQ).  Outcomes related to implementation are educators’ self-efficacy about EASEL practices (as assessed on the Educator Self-Assessments) and dosage of the EASEL practices (as assessed through the EASEL Fidelity measure).  We have attempted to clarify this in the manuscript (see Abstract p.2-3 and Objectives p.8). We have also added more about our rationale for a type 2 hybrid study in the Study Design section (p.9-10). |
| 2 | Following the first question, it is unclear how the sample size calculation was done. Which outcome it was based on? “25% difference in child outcomes” refers to which outcome? Since it is a cluster randomized trial, how the clustering effects (e.g., what were the assumed ICCs or variance components) were considered in the sample size calculations? I notice that there are several places (pages 8 and 23) were mentioning the sample sizes. I would like to suggest to have a “sample size and power” section to (at least) include the following information a. Outcome used for power calculation b. Assumed effect size c. Cluster size d. Number of clusters e. Assumed ICCs (or other measure of clustering effect) | We based our power calculations on the effects on educator’s teaching outcome and children’s social-emotional outcomes, commonly reported outcomes amongst universal classroom-based educator-delivered interventions similar to ours (e.g., Luo et al., 2020; Landry et al., 2014; Flook et al., 2015). We have since done more power calculations based on the primary outcome of the educator’s classroom environment and practices and have elaborated on this section with more details (p.29).  We have also accounted for cluster sizes (i.e., 3 educators per centre, 45 children per centre) and have detailed the ICCs used on p.29. |
| 3 | A more detailed description of statistical analysis is needed. There seems to be many outcomes measured. Will all of them be analyzed using MLMs. What kind of MLM models the authors is planning to use. Will the authors use the change scores as the outcome, or an ANCOVA-like approach will be used? Will the analyses be unadjusted (since there is no covariates-adjustment mentioned in the section)? | We have expanded on the Quantitative Analysis section (p.28-29) to provide the requested information about the models. |
| 4 | If there are multiple co-primary outcomes, adjustment for multiplicity is needed | We have edited the manuscript to specify that there is only one primary outcome (i.e., educators’ teaching practices and classroom quality as observed on the ECCOM). Thus, an adjustment for multiplicity is not needed since we do not have co-primary outcomes. |
| 5 | There are many places with “Error! Reference source not found”. Please check the links inserted in the manuscript. | There were errors in converting the Word document to PDF when we submitted it to PLOS ONE. We have since fixed these errors. |
| **REVIEWER 2** | | |
| 1 | The study title requires further specification. What outcomes exactly? Are we examining social emotional competence and learning outcomes? Please be specific. | In this study, we assess emotional and behavioral regulation, prosocial skills, and executive functioning skills. Due to the word limit, it is unfortunately not possible for us to specify all the study outcomes, but we have modified the title to specify “social-emotional outcomes”, which generally covers the key study outcomes. This is defined more explicitly in the introduction of the paper (p.5). |
| 2 | “In an exploratory study, early childhood educators in Singapore ranked children's development of SEB skills as the most critical learning area in early childhood education. Yet, they also reported high professional development needs in their capacity to promote children's SEB skills along with other pre-academic skills (12). Taken together with findings from the Vital Voices for Vital Years 2 report (11), there is a need to expand efforts to improve early childhood educators' knowledge in developing children's SEB and EF as well as increasing educators' understanding of how to ensure their classroom practices are inclusive of children with diverse needs” - much of the information presented in the introduction is highly localized, suggest rephrasing and making it more succinct to make it reader friendly for a wider audience | Thank you for the suggestion. Per the reviewer’s suggestion we have amended the Introduction (p.5-6) to help readers understand how the work fits within the international context. That said, the issues raised in the Introduction have been of particular interest to educators and policymakers in Singapore in recent years, therefore, we believe we it is important to provide some background to help contextualize the information for readers. |
| 3 | Although the authors gave some reasons to support the choice of the EPIS (Explore, Prepare, Implement, Sustain) Framework, it is unclear why this framework was chosen over others, e.g. the dynamic sustainability framework. In my reading of the manuscript, this seems like a viable choice given that interventions are not static and can be continually improved. What is the main outcome of the study? | The main goal of this study is to evaluate effectiveness and implementation. The EPIS Framework is a good fit for these goals because it informs how to evaluate the implementation of the EASEL Approach. Specifically, we are using the EPIS Framework to guide our evaluation of the impact inner and outer context factors exert on implementation outcomes, particularly during the Explore and Prepare phases of the implementation, as part of a hybrid effectiveness-implementation trial. Finally, since our main focus is implementation, with only a minor focus on sustainment, we did not consider the Dynamic Sustainability Framework a good fit for this stage of our work. |
| 4 | Several instances of “Error! Reference source not found” throughout the manuscript. Sloppy. | There were errors in converting the Word document to PDF when we submitted it to PLOS ONE. We have since fixed these errors. |
| 5 | “Multilevel Models (MLMs) will be used to assess differential patterns of change over time in each effectiveness measure between intervention and control groups across baseline and later timepoints while accounting for and measuring the effect of nonindependence of individual subjects (i.e., educators nested within childcare centers; children nested within both classrooms and childcare centers)” - this is very broad and generic statement. What MLM models exactly? Are you dealing with ‘aggregated’ or ‘collapsed’ variables? Scaled weight analyses or unweighted analyses? Further elaboration and details are missing here. It is prudent to state and adjust for foreseeable confounders in the final analysis. | We have expanded on the Quantitative Analysis section (p.28-29) to provide more details. |
| 6 | “Data will be stored on a bespoke database run from a locally-hosted, secure server that is accessible only to CHILD researchers” - what exactly is this? Is this something created specifically for the purposes of the study? | This is REDCapCloud which the National University of Singapore hosts. Only CHILD researchers have access to the EASEL trial database. We have since removed the word ‘bespoke’ from our manuscript to avoid confusion (p.31). |
| 7 | Would the authors have close monitoring of attrition and adjust the sample size accordingly? | We will closely monitor attrition and adjust the sample size accordingly before the intervention commences (i.e., before educator training is provided). However, we may be unable to replace educators who withdraw after the intervention has been initiated (and the children they teach) due to logistical reasons (e.g., replacement educators would not be able to attend a full-day training and receive coaching for a shorter period). |
| **REVIEWER 3** | | |
| 1 | p. 3: The contribution of this study to the literature is not yet fully clear. Only the last bullet point seems directly related to ’contribution to the literature’. The authors should indicate here: what will be the new knowledge after completion of this study? | We have amended these bullet points to provide more information about contributions to the literature (p.3-4). |
| 2 | Abstract, Introduction, Discussion: I doubt whether an ECEC program can ‘equip’ 3-6 year old children with competences and skills. ECEC programs can stimulate children’s development and skills at an early age and these skills are significant precursors of more holistic, broader skills at elementary school. I only suggest to use vocabulary that is developmentally appropriate, i.e. fits in with insights from developmental psychology and educational science. | An important component of learning in early childhood settings is school readiness skills that help prepare children for success in elementary school (and beyond). We therefore believe that important skills and competencies are taught in early childhood settings. That said, it is important not to overstate what can be accomplished in early childhood settings. Thus, in response to the Reviewer’s comment, we have reviewed the manuscript and tried to ensure that the language fits with insights from developmental psychology and educational science (p.2, 5, 33). |
| 3 | p. 4: What does ‘inclusive’ mean in the context of this manuscript? The word ‘inclusion’ has been used in various ECEC contexts (e.g., immigrant families, children with autism, challenging but normal behaviour of toddlers, etc.). The manuscript suggests that certain children are challenging for ECEC teachers in Singapore. However, in the design, sample, or analysis, there is no explicit link to certain groups of children (see also below). | The EASEL Approach is intended to be a universal classroom intervention and meant for children of varying abilities (e.g., children with developmental disorders, learning difficulties). It is not targeted to specific groups of children. Therefore, educators are taught and coached on how to apply and tailor the EASEL practices to all children in their classroom. We have amended this section to reflect this (p.6). |
| 4 | Table 1: The authors describe the selection process for the 9 EASEL themes with detail (see Table 1). Could they also report the scores for criteria 1, 2 and 3? Which instructional behaviors are still considered challenging for many ECEC teachers in Singapore? | To clarify, there were no scoring processes for shortlisting the 9 EASEL practices. Rather, we used procedures established in the literature to select the EASEL practices – i.e., consultation with experts. We consulted with experts at a national tertiary institute for early childhood educators in 3 meetings. In these meetings, we asked our partners to identify the EASEL practices that early childhood educators would benefit the most from learning. The process for selecting the EASEL practices will be described in detail in a manuscript currently in development. |
| 5 | p. 6: This question may be somewhat difficult, but I would like to know the answer. There is a new intervention that is not grounded with a specific theory of framework, but that seems to be designed in a systematic but also eclective approach. What do the 9 selected themes from the EASEL intervention represent? Is it a bottom-up selection by an expert committee and/or is it a selection of themes that are generally inadequate in regular ECEC in Singapore? Is it a coherent set or a heterogeneous blend of themes, is it a list of 9 separate items or is there perhaps a further categorization possible? For example, are the themes mostly related to instructional support or is it related to instructional support, classroom organization and/or emotional support? Or using a different categorization, which themes are linked with SEB and which ones with EF (see p. 3 for these abbreviations). And can the set of 9 themes be trained in a similar instructional format? | The EASEL Approach was developed using common elements approach, a framework that is becoming more popular in the literature (see References 17-22 of the manuscript).  In brief, a common elements approach involves systematically searching the literature to identify effective interventions for target outcomes. It is then followed by a process of extracting practices (i.e., elements) from intervention materials (e.g., manuals, and accompanying programme resources). Once elements have been extracted from all the identified interventions, elements common across the identified interventions are noted. This was the process we undertook to develop the EASEL Approach, described in a manuscript currently in development.  After the common elements were identified, we selected a final list of 9 EASEL practices from the complete list of 25 practices. This selection process was undertaken through consultation with external and internal subject matter experts for the reasons listed in the manuscript (p.14). The consultation focused on two factors: (a) the feasibility and effectiveness of the common elements and (b) how the common elements should be combined into a larger package (e.g., a balance of antecedent and consequential practices).  Table 1 lists the four outcome domains that each EASEL practice targets from the literature.  Out of the EASEL Practices listed in Table 1, seven are practices that can be employed in everyday educator-child interactions (e.g., Active Listening, Providing Choices) and support the development of SEB+EF skills throughout the school day, whereas two practices are designed to be embedded by educators in their curriculum to promote SEB+EF skill development (e.g., Dramatic Play, Games Promoting EF). When put together as a package, the EASEL practices are intended to help educators learn new instructional practices that help promote SEB skills, improve classroom organization, and improve teacher-student relationships. These goals are reflected in the outcomes assessed in the trial. |
| 6 | p. 7: using A non-blinded à a (i.e., without capital) | We could not find this error in the manuscript – can the reviewer be more specific? Thank you. |
| 7 | p. 9, Design & sample: The authors do not mention attrition at center, teacher or child level. Is this realistic? | We appreciate the Reviewer raising this point. We anticipate that there will be attrition throughout the trial. It is likely that attrition will be at the teacher or child level and not the centre level. We now discuss attrition in the paper and how it will be addressed. |
| 8 | p. 13: preparation has occurred à I am not a native speaker, but is this grammatically correct? | Yes, this is grammatically correct. |
| 9 | p. 14: naïve à no “ï”? | ‘Naïve’ is the correct spelling. |
| 10 | p. 15-19: In this part, there seems to be some redundancy. The implementation theme is repeated in this part of the text. Could the authors write this part in a more concise way / with less repetition? | We have attempted to make this section more concise (p.15-18, 20-21). However, we retained some of the text as it illustrates how the EPIS framework was applied to the current study. |
| 11 | p. 17: What are the current Covid19-restrictions in Singapore ECEC centers? | There are currently no restrictions; however, depending on the global and local COVID situation, previous COVID-related restrictions may be re-instated (e.g., no external visitors, including research staff, allowed into centers). This would affect our ability to conduct in-person coaching, and we would have to pivot to online coaching instead. We have previously experienced the need to very quickly modify and pivot research trials during the pandemic and, therefore, now have contingency plans in the rare instance that we may need to move to more remote data collection methods. |
| 12 | p. 20: The authors have selected the ECCOM measure (Early Childhood Classroom Observation Measure), but readers cannot accurately judge from this design paper whether / why this is a good choice. This is, first of all, relate to my abovementioned comment related to the exact content of the EASEL intervention, which was not fully clear to me. Second, the authors indicate which subscales from the ECCOM are not selected, but the reader cannot find which subscales from the ECCOM are considered relevant in this evaluation of EASEL. | Table 5 details the subdomains that we are using in this trial. We have also provided in Table 5 a list of how the ECCOM subdomains map onto the EASEL practices. As such, the ECCOM is well-suited to assessing whether the classroom environment and teaching quality have changed due to the EASEL Approach. Furthermore, the ECCOM is freely available and thus can more easily be used by administrators compared to other classroom observation measures (e.g., CLASS). As a result, we thought that the ECCOM could be factored into later sustainment and scaling-up plans if appropriate. We have amended this section to state this more clearly (p.23). |
| 13 | Are the ECCOM raters (i.e., the CHILD researchers from the Centre for Holistic Initiatives for Learning and Development) blind to experimental condition? Or are they relatively close to the research team and/or the EASEL intervention so that they are aware and may recognize the location and intervention? | Not all ECCOM raters were naïve to the experimental condition (i.e., 50% of raters were naïve). This decision was made for pragmatic reasons (e.g., insufficient staff who were blind to study conditions). |
| 14 | If random assignment is “fluked” in this relatively small sample (i.e., at center level), what will be the strategy of the researchers? Will they include covariates? Will they apply other statistical analyses to control for possible confounding? | If differences in baseline variables suggest that randomization was not successful, we will control for baseline differences in our analyses. |
| 15 | p. 23: Could the authors give an indication of the design effect (ICC) for teachers and children? How does this affect the statistical power of their analysis? | As per the literature, the ICCs for educators and children vary around 0.2-0.3 (e.g., Landry et al. 2014; Flook et al., 2015). We have factored these into our statistical power calculations (p.29). |
| 16 | p. 23: Could the authors qualify the a priori assumed difference of 25%: is this a small, medium or large effect according to the rules of thumb of Cohen (1988)? The authors could actually base their sample on effect sizes from previous experimental studies into this domain. The challenge is that meta-analyses have demonstrated small-to-medium differences at process quality/teacher/center level, whereas the experimental effects for children seem generally smaller (see, for example, Egert et al., 2018). I can imagine that statistical power is not the same for the research questions at teacher level (a) and at child level (b). | Thank you for this suggestion. We have since used the effect sizes for our primary outcome (i.e., educator teaching practices) from Luo et al. (2020), Landry et al. (2014) and Flook et al. (2015) publications in our power calculations (p.29). |
| 17 | p. 23: The proposed multi-level analysis takes into account that educators are nested within childcare centers. However, the number of centers and, relatedly, the number of teachers seems relatively small. Perhaps the authors could consider to include teachers as a factor which may be explored in a preliminary analysis (instead of a level). | Thank you for this suggestion; this is a good point. We will review this at the end of the trial and depending on attrition rate and the resulting statistical power, we may need to do this instead. |
| 18 | p. 23: See my previous comment: The authors introduce the importance of challenging behaviors of some students in an inclusive ECEC setting. I had expected therefore that the analysis would include a child factor that would capture this child characteristic instead of a universal, but perhaps too general effect for all children. | The EASEL Approach primarily targets educators’ teaching practices which are likely to promote children’s development of social skills, emotional regulation, positive behavior and executive functioning skills. These practices are not only targeting the reduction of challenging behaviors. As a result, our outcome measures assess children’s social skills, emotional regulation, behavior and executive functioning. |
| 19 | p. 23: The authors emphasize the importance of implementation. Do I understand correctly that implementation is not a moderator in their analysis? If so, I suggest that a continuous moderator seems vital for the analysis of a newly developed and implemented intervention? I emphasize the continuous nature of such a variable, because I read that ‘why there were of were not changes’ as an all-or-nothing matter. | One of our implementation outcome measures is an observational assessment by coaches to assess how often each EASEL practice is being implemented in the classroom. This is completed in the first month of coaching, mid-intervention and post-intervention. Data from this measure is continuous and will be factored in as a mediator in our analyses. We have added this information to p.29 for more clarity. |
| 20 | p. 24: Qualitative - or: Qualitative analysis | Thank you, we have amended this for the quantitative and qualitative sections (p.27 & p.29). |
| 21 | p. 24: The thematic content analysis is not described with adequate detail. The authors could describe the qualitative analysis with the general methodological approach, a priori themes in a coding scheme, use of software, description of coding and training coders, qualitative analysis after coding (etc.). | This section has been rewritten for clarity and more information has been provided about the qualitative analysis (p.29-30). |
| 22 | p. 24: The triangulation and mixed methods analysis is not described with adequate detail. Triangulation suggests that two different types of data (i.e., qualitative & quantitative) are related to a similar construct (i.e., implementation of EASEL). My question is: is there a order in the analysis? Which analysis may follow and build upon the other? And my other question is: what will the researchers do when the different types of data do not converge? Terminology like convergent / parallel / embedded / sequential / exploratory vs. explanatory may give a more accurate picture of the planned analysis. | This section has been rewritten for clarity and more information has been provided about the specific approach to mixed methods analysis (p.30-31). |
| 23 | By the way, there were some missing links missing (“Error!”) in the manuscript (e.g., p. 12 and 14). | There were errors in converting the Word document to PDF when we submitted it to PLOS ONE. We have since fixed these errors. |
| 24 | A final note: I take the liberty to share some of my thoughts related to the intervention and its implementation. The authors indicate that a kick-off with the research team and a one-day workshop is the starting point of the intervention in practice. Coaching will be the next phase and the authors indicate that they want to identify barriers and challenges with the implementation based on meetings with ECEC teachers.  My thoughts / concerns are as follows:   - There is no (small-scale) pilot. Hence, learning about barriers and challenges takes place during the evaluation with measures at teacher and child level. - A one-day workshop seems very brief, even it is one-on-one. - The coaching seems very helpful and, in my opinion, is seriously needed for effective implementation at teacher level. The authors refer to Elek and Page (2019), which is an important source. Also other reviews have (recently) been published related to coaching in an ECEC context (e.g., Blewitt et al., 2011; Donath et al., 2023; Egert et al., 2018; Obee et al., 2022; McLeod et al., 2023; Young et al., 2021). These new sources are interesting for the readers of this paper (a) and they may provide more guidance in the design and delivery of the coaching part of the newly developed intervention (b). - I hope that the research team will have constructive dialogues with the teachers as experts in their own classroom and with their students.   I have added these comments from my background in ECEC pre-service and in-service training and other forms of professional development, including coaching, and I would like to understand this new intervention a little bit better. The question behind this question (i.e., my concern) is that it may be very difficult for the research team, the instructor in the one-day session, the coach in the period thereafter, and, most of all, for the ECEC teacher – after a relatively brief training – to implement various changes in his/her behavior throughout the day in relatively large classrooms with young children. To be perfectly clear, my questions are linked to positioning the intervention for readers of the journal, and they may also be interpreted as motivations for this study. | We thank the reviewer for detailing their thoughts and suggestions for us; it is much appreciated. We attempt to respond to some of the comments here.  Although there was no small-scale pilot, we consulted with our partners (e.g., university lecturers of early childhood educators) to refine the EASEL Approach, contextualize it for early childhood educators in Singapore, and ensure the implementation strategies we used were appropriate and did not pose undue burden on educators involved in the trial. For example, there was much discussion with the early childhood operators about how long training should be, as well as the childcare centers' time and resource constraints, which resulted in the decision to hold a one-day workshop.  We have noted the papers the reviewer has suggested on coaching and will review these as well. Thank you! Our current approach to coaching is collaborative and recognizes educators as the experts in their classrooms and with their students. We focus on building rapport with the educators during the training workshop and in the first weeks of coaching before moving on to work with educators to implement and incorporate the EASEL practices into their everyday interactions. Our approach to coaching (including duration, frequency and structure) was selected to overcome some of the barriers to behavior change that may exist due to the relatively brief training and unfamiliarity of educators with the EASEL practices. |
