# Supplementary material for: Assessing the effectiveness and implementation of a universal classroom-based set of educator practices to improve preschool children’s social-emotional outcomes: Protocol for a cluster randomized controlled type 2 hybrid trial in Singapore
Source: PLoS One. 2023 Sep 20;18(9):e0291723. doi: 10.1371/journal.pone.0291723 (PMC10511079; doi:10.1371/journal.pone.0291723)
Supplement: Attachment — Submitted filename: PONE-D-23-00745 Response to Reviewers Aug2023.docx [file pone.0291723.s004.docx]

PLOS ONE Manuscript Response to Reviewers

ID: PONE-D-23-00745

Title: Assessing the effectiveness and implementation of a universal classroom-based set of educator practices to improve preschool children's social-emotional outcomes: Protocol for a cluster randomized controlled type 2 hybrid trial in Singapore

**We thank the reviewers for offering constructive and helpful comments to help improve our manuscript.**

**Please note that the page numbers referred to in the authors’ responses are in reference to pages in the manuscript with tracked changes.**

| **#** | **Reviewers’ Comments** | **Authors’ Response** |
| --- | --- | --- |
| **REVIEWER 1** | | |
| 1 | On page 24-25, the authors mentioned "Random intercepts and slopes by subject (e.g., child or educator) will be tested and used, if necessary. Otherwise, we will use fixed effects to conserve statistical power." It seems to be a bit vague to me. Can the authors be more specific on this? What does it mean "if necessary" and when the fixed effect model will be applied. | Thank you for your comment. We have amended this section to clarify what we meant (p.24). |
